# Healthcare and Welfare Policy Efficiency in 34 Developing Countries in Asia

**DOI:** 10.3390/ijerph17134617

**Published:** 2020-06-27

**Authors:** YongChan Kim, Min Jae Park, Erdal Atukeren

**Affiliations:** 1 Department of Business Administration, Seoul School of Integrated Sciences and Technologies (aSSIST), Seoul 03767, Korea; 2 Department of e-business, Ajou University, Suwon 16499, Korea; geglove@ajou.ac.kr; 3 Business School Lausanne (BSL), CH-1022 Chavannes (VD), Switzerland; erdal.atukeren@bsl-lausanne.ch

**Keywords:** data envelopment analysis, healthcare investments, efficiency of investments, Asian economies, healthcare policy, economic development, health economics, human capital

## Abstract

The healthcare and welfare policies of nations, as well as the amount of investments put into these areas, vary across countries. Investments in healthcare and welfare have been increasing worldwide which brings the question of assessing the efficiency of these investments. There are, however, difficulties in evaluating the effectiveness of such investments due to differences in countries’ economic development levels and due to the differences in data definition issues. There are only a limited number of studies in the literature that employ consistent and comparable indicators across countries. This study evaluates the healthcare investment efficiency and health competitiveness efficiency of 34 developing countries in Asia using a two-stage dynamic data envelopment analysis approach. Furthermore, we employ a broader measure of indicators on national healthcare and welfare policies and outcomes, in addition to the investment data on healthcare and welfare expenditures. Our findings indicate that the establishment of an investment environment with a consolidated approach and management is an important factor that increases the efficiency of investments in healthcare and welfare sectors. A consistent delivery of the national policy strategy is also crucial for reaching the medium-and long-term targets for each country. For example, if a country establishes healthcare and welfare policies that focus on improving its indicators with low efficiencies, the output will be improved and a better return on investment will be ensured in a long-term perspective.

## 1. Introduction

The World Health Organization (WHO) stated that one-quarter of global deaths, including one-third of child deaths, are related to environmental factors that can be improved [1]. In countries with low economic levels, environmental factors significantly contribute to the incidence of disease and death. In many developing countries, economic policies are rather geared towards achieving higher real economic growth rates; and policies that tackle environmental challenges in this process are relatively neglected [2]. 

Asia includes the adjacent islands of the continent, the Indian Ocean and the Pacific, and the Asian continent accounts for approximately 60% of the world’s population [3]. Depending on the level of development, countries in Asia are subject to air pollution arising from increased urbanization, low-grade fossil fuel use, indoor air pollution from biofuels, heavy metal pollution from mine development, water pollution from inadequate sewage and wastewater treatment facilities, and other chemical pollutions. Infectious diseases caused by climate change, sea-level rise, soil degradation, and spread of infection through animals are also increasing throughout Asia [4]. As such, people living in Asian countries are facing health problems and risks due to the influence of worsening air, water, and soil quality. The extent of these problems, however, depends on the individual countries’ geographic, economic, and cultural characteristics [5].

Governments set the investment directions for healthcare and welfare systems through policies and regulations. Such policies and regulations have consequences at both national and societal levels. Investing in healthcare and welfare systems is not only aimed at saving lives but it is also an important investment for the national economy [6]. This is because a healthy population and a well-designed welfare system can improve the productivity of the human capital, which can have a positive impact on national competitiveness [7]. As the budget for healthcare and welfare investments are usually constrained within the government’s overall budget system, an investigation of the efficiency of investments in healthcare and welfare is essential [8]. It should be noted that, strictly speaking, healthcare and welfare policies would be different from each other. However, the “healthcare and welfare” terms are generally used together since healthcare and welfare variables are related to each other and integrated. As a result, treating them separately might lead to difficulties in evaluating the effects of these policies.

Against this background, this study evaluates the characteristics and effectiveness of national healthcare and welfare policies in 34 developing countries in Asia using dynamic data envelopment analysis (DEA) techniques. The healthcare and welfare policies of developed, developing, and least-developed countries in the world possess different characteristics. The share of investment in healthcare also varies from country to country [9,10]. The share of investment in healthcare and welfare has been increasing worldwide in line with the “Millennium Development Goals (MDGs)” and “Sustainable Development Goals (SDGs)”. Nevertheless, there are difficulties in evaluating the efficiency of such investments due to different economic development levels, different external environments, and domestic policy differences in each country [11]. Due to these factors, there are only a few studies in the literature that have employed consistent and comparable indicators across countries. Most of these studies focus on developed countries.

In order to assess and compare the efficiency of healthcare and welfare investments by different countries, it is first necessary to use compatible data on healthcare and welfare expenditures using the same standards for each country in the study [12]. There are five priority areas suggested by key health policy experts from the World Health Organization (WHO) and Non-Governmental Organizations (NGOs) [13]. The variables that cover these priority areas would form a compatible and comparable basis for cross-country comparisons on the efficiency of healthcare and welfare investments. These priority areas are the following:

1. Universal Health Coverage
2. Primary Health Care
3. Health Systems Strengthening
4. Health Financing
5. Access to Medicines

In our study, the “Universal Health Coverage” and “Primary Health Care” indicators are associated with one of the input variables (“Current Health Expenditure (CHE) per Capita in PPP”) in our two-stage DEA model. The “Health Systems Strengthening” category is the main goal of this study and it is linked to enhancing national competitiveness. The “Health Financing” category is related to investments and national healthcare and welfare policies as the second input variable in our TS-DEA model, proxied by “Domestic General Government Health Expenditure (GGHE-D) as % of Current Health Expenditure (CHE)”. Finally, the priority area “Access to Medicines”, is associated with an indicator such as healthcare personnel that includes physicians, pharmacists, nurses, and midwives and it is included in the intermediary stage of the analysis.

In addition, the efficiency comparisons on healthcare and welfare investments might also be sensitive to the methods used. There are a multitude of methods for measuring the characteristics and efficiency of healthcare and welfare policies by different countries [14]. One method that comes to the fore in the literature is the “Data Envelopment Analysis (DEA)” technique. Hence, we employ the dynamic DEA technique in this study.

Compared to previous studies that use data on investment expenditures in the healthcare and welfare sectors only [15], our study employs a broader range of data on healthcare and welfare expenditures and more detailed national health indicators. We include the incidence of tuberculosis, life expectancy, and under-five mortality rates in our study, in addition to the conventional data on healthcare and welfare expenditures. As such, our study is designed to be more comprehensive in comparing the efficiency of the healthcare and welfare policies of the countries in our sample. Earlier studies argued that as real GDP increases, a country invests more in healthcare and welfare programs and conducts more elaborated healthcare policies [16]. However, in terms of efficiency, a higher economic development level does not necessarily bring about more efficient healthcare policies [17]. Therefore, the importance of having systematic healthcare and welfare policies/programs under limited financial and physical resources is emphasized [18]. Our paper stresses the need for continuous study on the utility of such investments in the healthcare sector in each country and highlights the need for collaboration between all the stakeholders involved, which is related to both efficiency and cost/benefit perspectives. Both the methodology and the operationalization of input and output variables are different than earlier studies in the literature, providing a new perspective on measuring the efficiency of healthcare and welfare investments. Despite the importance and urgency of the topic for Asian countries, the literature lacks comprehensive and consistent studies in healthcare and welfare investment efficiency evaluation for developing Asian countries. Our study hopes to help fill this gap.

The rest of the paper is organized as follows. In Section 2, we provide the theoretical background and the characteristics and efficiency of healthcare and welfare policy and review the literature. In Section 3, we discuss the research design and the methodology. We use the two-stage DEA technique, which provides a more dynamic analysis framework for the variables in the model compared to the conventional static DEA approach. The data used in this study are obtained from public data sources such as the World Health Organization (WHO), the United Nations (UN), and the World Bank (WB). In Section 4, we present and discuss our findings. Section 5 discusses the policy implications of our results and concludes the paper. Finally, in Section 6, we acknowledge some shortcomings of the study due to data availability constraints and suggest directions for future research in the field.

## 2. Review of Literature and Latest Trends

### 2.1. Literature Review

The literature on the efficiency of the public sector focuses on public sector performance (PSP) and public sector efficiency (PSE). In this section, we discuss in more detail the general findings in the literature that suggest a further potential for increasing the efficiency of public sector investments. However, the results in the literature are rather broad in scope and not specific to healthcare and welfare sector investments. In earlier studies, only an index of public expenditures on healthcare was used as a comparison indicator in the healthcare sector. A more accurate assessment of the efficiency of investments in healthcare and welfare sectors requires the use of more specific and detailed (sub-) indicators [19].

Afonso et al. (2005) employed the non-parametric frontier production function approach using a composite index and seven sub-indices to examine input and output efficiencies of public healthcare spending in 23 OECD countries. The findings indicated the existence of large differences in the public sector performance (PSP) and public sector efficiency (PSE) across the countries in the sample, suggesting an important potential for expenditure savings in many countries. It should be noted that an increase in private healthcare spending does not necessarily bring about improvements in the public finance and healthcare system. A more systematic approach is needed to enhance the sustainable healthcare structure from a national perspective [20]. In addition to the public healthcare sector, comparative studies on the efficiency of public and private hospitals have been conducted. Jing et al. (2020) used data envelopment analysis (DEA) and propensity score matching (PSM) to measure the technical efficiency. This study suggested that public hospitals should strive to improve standards of management and focus on the structure management of human capital in hospitals such as health care providers as well as cost reduction of hospitalization. In addition, private hospitals are recommended to expand in size through an appropriate restructuring [21].

Spinks and Hollingsworth (2009) provide a cross-country comparison of technical efficiency in health production using health expenditure data and socioeconomic indexes such as education, employment, and GDP per capita as input variables. The output variable is chosen as life expectancy (in years) at birth. The study has limitations in comparing healthcare production efficiency in a broader setting of input and output variables [22]. Further studies in the literature include Evans (2001) and Woolcock (2018). Evans (2001) estimated the efficiency of a national health system in 191 countries using the health expenditure index as an input variable [23]. Woolcock (2018) also compared the relationship between gross national income per capita and disability-adjusted life years (DALY) in developing countries [24].

As reviewed above, the limited number of earlier studies in the literature on national healthcare and welfare policy efficiency used a traditional input variable such as health expenditure and did not characterize output with a variety of variables that capture different aspects of healthcare and welfare policies. In addition, from a methodological perspective, there has not been many studies that include a dynamic time frame concept. These limitations lead to difficulties in comparing the changes in efficiency overtime, which is an important aspect in policy decision-making and policy/program evaluation. A systematic evaluation of the changes in the efficiency of investments is an important dimension in establishing national healthcare policies, implementing the relevant strategies, and validating the achievement of national objectives in these areas.

### 2.2. Latest Trends in Health and Welfare Policy

The G20 Summit, launched in 2008, includes 20 major countries of the world based on the size of their economies. The Health and Finance Ministers’ Meeting under the G20 Summits began in 2017. In June 2019, the leaders at the G20 summit in Japan acknowledged that cooperation between health and financial authorities could be a driving force for improving the efficiency of health finance in developing countries. A testimony is made for the fact that the healthcare policy is closely related to a country’s limited financial situation. The meeting featured a key agenda on achieving sustainable economic growth through universal health coverage in developing countries. The detailed agenda includes (1) commitment of the Minister of Health and Finance with understanding on the importance of Universal Health Coverage (UHC) financing in developing countries, (2) a description of proposals and best practices for cooperation with health and financial authorities, and (3) suggestions for strengthening cooperation between the World Health Organization (WHO) and World Bank (WB) [25]. Since the establishment of a quality and sustainable health care system depends on both health and financial authorities of each country, it should be recognized that it is important to create a policy framework for sustainable cooperation in the design process and procurement of health care systems.

To date, there has been a continued interest in cross-national comparisons of health systems and policies between policy analysts and policymakers. Research in the field of healthcare and welfare has expanded over time, but a systematic assessment of the quality of these studies received less interest. Perhaps the concept of “quality” itself is multi-dimensional and can vary from environment to environment. There have been assessment tools for some of these studies or methods, but they are not designed for cross-country comparisons of health systems and policies. Research on health systems and policies also suggests that differences in methodological approaches might be important for the results obtained [26].

Every country recognizes the importance of its healthcare and welfare systems/programs, and thus investment in these areas continues to increase: Improving the health and welfare of the people is a key national policy objective. This is a challenge for most OECD countries, along with the growing proportion of government debt, when viewed in conjunction with the financial sectors of the OECD countries’ economic and financial crises. In particular, the 2010 OECD report suggests that public spending on healthcare is one of the largest government spending items, with an average of 6% of GDP. The increasing costs associated with the healthcare sector correlates with the aging of populations and the development of medical technology [27]. Some studies project national health expenditures to account for up to 19.4% of GDP by 2027 [28].

Therefore, improving efficiency is essential to meet rapidly increasing medical needs and to maintain investment and systems while determining the operation and direction of public finances in these health and welfare sectors [29]. The question is what should be done to improve the efficiency of healthcare and welfare sectors? One option is to use a comparative perspective and examine the performance of various healthcare and welfare systems with reference to their strengths and weaknesses. Such country-specific comparisons could lead to policies that could improve the effectiveness of overall healthcare and welfare systems. This is also contingent on the maintenance of good indicators and improvements made in each country’s health care systems.

Trends in the Asia-Pacific (AP) region show that, in recent years, we have been intensively investing in the areas of health infrastructure, health governance, and health financing. In particular, the Asian Development Bank (ADB) announced its “Health 2015–2020 Operation Plan” in June 2015, which suggested the initiation of strategic investments in developing countries in the areas of health-related infrastructure, health governance, and health financing to improve Universal Health Coverage. In particular, the ADB is expanding its public and private health services through its “Operational Plan for Health (OPH)”, in collaboration with the United Nations (UN).

Similarly, improving the outcome efficiency for health sector investments in the Asia-Pacific (AP) region is an important policy objective along with increasing investments in integrated and cost-effective programs. The objectives include the improvement and expansion of healthcare in low- and lower-middle income countries, strengthening new and innovative ways in current policies, and ensuring the quality health care that maintains efficiency and cost-effectiveness [30].

The current trends and developments on the characteristics and policies of the healthcare and welfare sector in the Asia-Pacific region and around the world point to a great deal of interest for efficient use of investments in healthcare and welfare areas, and this necessitates a systematic analysis of the policy and program outcomes at the national level. This study aims to contribute to the literature by assessing the changes in the efficiency of healthcare and welfare investments in 34 Asian developing countries between 2002 and 2016. Most of the countries in our study are classified as low- or lower-middle income countries, with the exception of some oil-producing countries in the Middle East.

## 3. Materials and Methods

### 3.1. The Two-Stage DEA Approach

The concept of efficiency is used for evaluating the outputs or outcomes that result from the use of resources in the performance of an organization or country to accomplish its objectives [31]. Efficiency means “the ratio of output to input” [32,33]. To measure efficiency in the healthcare and welfare investments, this paper uses the DEA analytical method that was first proposed by Charnes, Cooper, and Rhodes (1978) [34].

DEA is a non-parametric approach based on linear programming, with the Charnes, Cooper, and Rhodes (1978) model (CCR) that assumes constant returns to scale (CRS) [34], and the BCC model which deals with variable returns to scale (VRS) proposed by Banker et al. (1984) [35]. In this study, we perform an output-oriented DEA analysis that maximizes the level of output elements while maintaining the level of inputs. The output-oriented DEA model is useful in the efficiency evaluation of port, steel, and automobile industries where the input factor is large fixed capital goods [36]. We use the output-oriented DEA analysis in this study because of the large nature of the capital requirements in the healthcare and welfare areas.

**Figure 1:**
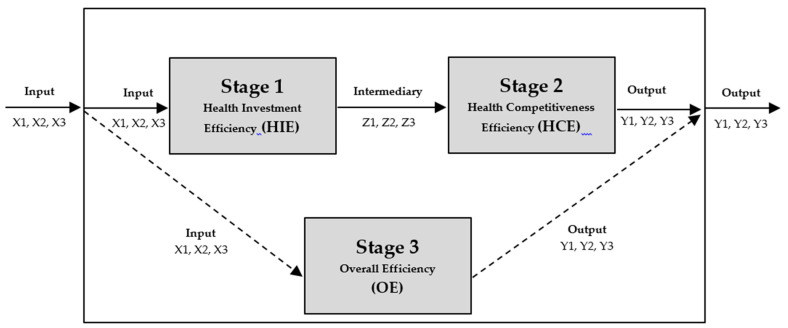
Two-stage data envelopment analysis (DEA) structure of the study.

On the other hand, the traditional DEA model measures the relative efficiency of each decision-making unit (DMU) with multiple inputs and multiple outputs. However, the process by which input elements are converted to output elements is not clearly explained. This is an advantage of the existing DEA model, but also limits the application of the DEA model in various areas. The Network DEA model was introduced by Färe and Grosskopf (2007) [37]. They first referred to the “black box” and focused on the conversion process inside the black box. They pointed out that the input DEA model was measured in the black box as an output element through a conversion process, and the actual conversion process has not been clearly explained. Therefore, they generalized the network model by focusing on the conversion process of the black box [38]. Tone and Tsutsui (2009) also identified the need for a new DEA model because a traditional DEA model could not belong to input and output simultaneously, since all activities can only belong to either input or output [39]. The following is the mathematical process used to analyze the two-stage DEA research model, as shown in Figure 1.

If we let $v_{i}$ denote the multiplier associated with input *i* (*I* = 1, 2, 3), $u_{r}$ denote the multiplier associated with output *r* (*r* = 1, 2, 3), and $w_{g}$ denote the multiplier associated with intermediate product *g* (*g* = 1, 2, 3), then, when calculating the system efficiency of decision making unit ${\left(DMU\right)}_{k}$, each process must comply with the frontier condition in that the aggregated output must be less than the aggregated input, which is the additional condition to the conventional constraints for the system.

(1) $$E_{k}=max\;u_{1}Y_{1k}+u_{2}Y_{2k}+u_{3}Y_{3k}$$

(2) $$v_{1}X_{1k}+v_{2}X_{2k}+v_{3}X_{3k}=1$$

(3) $$(u_{1}Y_{1j}+u_{2}Y_{2j}+u_{3}Y_{3k})-\left(v_{1}X_{1j}+v_{2}X_{2j}+v_{3}X_{3k}\right)\leq 0,\;j=1,\ldots ,n$$

(4) $$\left(w_{1}Z_{1j}+w_{2}Z_{2j}+w_{3}Z_{3j}\right)-\left(v_{1}X_{1j}+v_{2}X_{2j}+v_{3}X_{3j}\right)\leq 0,\;j=1,\ldots ,n$$

(5) $$(u_{1}Y_{1j}+u_{2}Y_{2j}+u_{3}Y_{3k})-\left(w_{1}Z_{1j}+w_{2}Z_{2j}+w_{3}Z_{3j}\right)\leq 0,\;j=1,\ldots ,$$

(6) $$u_{1},u_{2},u_{3},u_{4},\;v_{1},v_{2},v_{3},v_{4},w_{1}\leq \varepsilon$$

Constraint (3) conforms to the system and constraints (4) and (5) conform to the two sub-processes of the system, respectively. The additional constraints from the processes induce the relational Network DEA model stricter than the traditional DEA model. Once the optimal multipliers v_i, u_r, and w_g are calculated from the models above, the efficiencies of the three processes are obtained as:

(7) $$E_{k}^{\left(1\right)}=\left(w_{1}Z_{1j}+w_{2}Z_{2j}+w_{3}Z_{3j}\right)/\left(v_{1}X_{1j}+v_{2}X_{2j}+v_{3}X_{3j}\right)$$

(8) $$E_{k}^{\left(2\right)}=(u_{1}Y_{1j}+u_{2}Y_{2j}+u_{3}Y_{3k})/(w_{1}Z_{1j}+w_{2}Z_{2j}+w_{3}Z_{3j})$$

(9) $$E_{k}^{\left(3\right)}=(u_{1}Y_{1j}+u_{2}Y_{2j}+u_{3}Y_{3k})/(v_{1}X_{1j}+v_{2}X_{2j}+v_{3}X_{3j})$$

Thus, $E_{k}^{\left(1\right)}$ is used to calculate the efficiency of the first stage, $E_{k}^{\left(2\right)}$ is used for the second stage, and $E_{k}^{\left(3\right)}$ is used for the third stage.

### 3.2. Research Model and the Operationalization of Input, Intermediary, and Output Variables

Each variable in the model consists of relevant indicators obtained from public data sources, such as the World Health Organization (WHO), United Nations (UN), and World Bank (WB). In choosing the indicators to reflect the characteristics and efficiency of health and welfare policies in the countries in our sample, we have considered those that are most relevant and available for as many countries as possible. This approach is in line with earlier studies [40,41]. First, “Health Expenditure” indicators for the input variable included “Current Health Expenditure (CHE) per Capita in PPP”, which are most commonly used indicators in the assessment of the health and welfare sector. “Domestic General Government Health Expenditure (GGHE-D) as % of Current Health Expenditure (CHE)” and “Current Health Expenditure (CHE) as % of Gross Domestic Product (GDP)” were also included as measures of the government’s share of public investments in GDP.

**Figure 2:**
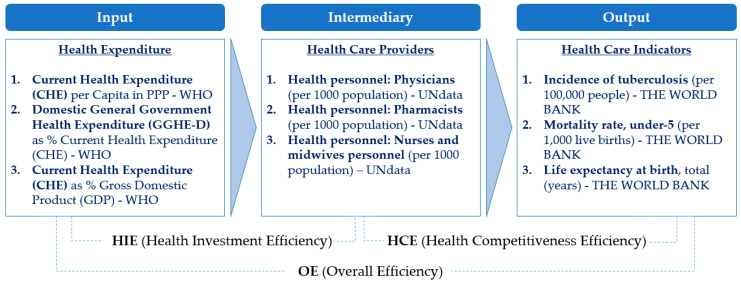
Research model: Efficiency of health care investment by country.

The second category was intermediary, which included indicators that were related to healthcare providers: Health personnel (physicians (per 1000 population)), (pharmacists (per 1000 population)), and (nurse and midwife personnel (per 1000 population)). Health personnel is important and requires management and training because as the national or government health expenditure increases, the number and proportion of healthcare professionals will increase. The third category was output, which included the incidence of tuberculosis (per 100,000 people), mortality rate, under-five (per 1000 live births), and life expectancy at birth, total (years). In particular, those indicators that are related to the output were selected because they can be used to compare the competitiveness of each country in the health, welfare, and medical sectors. For example, the incidence of the tuberculosis index has been rarely used in the research field of health and welfare, but it was judged to be useful in comparing the health infrastructural power of developing and developed countries. Similarly, the mortality rate and mortality under-five rates are comparable indicators of national competitiveness, and life expectancy at birth and total (years) are commonly used in the literature [42]. Therefore, to compare with the other two indicators, they can be used as complementary indicators. Figure 2 shows the structure and the variables of our model that will be used as the basis for the DEA analysis.

## 4. Results

This section presents the empirical implementation of the two-stage dynamic DEA approach using the theoretical model framework described in Section 3 and the calculation of relevant input, intermediary, and output variables, as shown in Figure 2. Our study includes 34 developing countries in Asia and assesses the efficiency of their healthcare and welfare investments for the time period between 2002 and 2016. The list of the countries and the data used in the analyses are included in the Appendix A.

We divide the data into three time frames and apply a three-years’ time lag as suggested by the results of previous DEA studies that have revealed that it takes about three years to derive results when any input is entered [43]. The first time frame is set as 2002~2004 for input 1, 2, 3, and 2005–2007 for intermediary 1, 2, 3, and 2008–2010 for output 1, 2, 3. The second time frame is set as 2005–2007 for input 1, 2, 3, and 2008–2010 for intermediary 1, 2, 3, and 2011–2013 for output 1, 2, 3. The third time frame is set as 2008–2010 for input 1, 2, 3, and 2011–2013 for intermediary 1, 2, 3, and 2014–2016 for output 1, 2, 3. Moreover, due to the nature of healthcare policies, the budget for medical care is more likely to be executed in a particular year than in other years. Hence, we used three-years’ average values for each variable. For example, “input 1” variable uses the average value from 2002 to 2004, “intermediary 1” variable uses the average value from 2005 to 2007, and “output 1” variable uses the average value from 2008 to 2010. Table 1 shows the organization of the data and the time frames using the example of Afghanistan. The same structure is used for all other countries in our study.

As it can be seen in Table 1, in Afghanistan, for example, three input variables corresponding to 2002–2004 were the input from TF_1 (Afghanistan1), and the efficiency values for three intermediary variables corresponding to 2005–2007 were measured. Efficiency values for three output variables (2008–2010) were measured from three intermediary variables (2005–2007). Similarly, the efficiency values of the three output variables (2008–2010) were also measured from the three input variables (2002–2004). Therefore, a total of three analyses was conducted on TF_1 for Afghanistan1. Since each of the three analyses was conducted for TF_2 (Afghanistan2) and TF_3 (Afghanistan 3), Afghanistan is calculated to have a total of nine analyses. As a result, since the efficiency analysis was conducted for a total of 34 countries, the total number of analyses in this study is 306 by multiplying 9 in 34 countries.

Our study includes the results of efficiency analysis across a total of three routes using the two-stage DEAs. The first analysis was the efficiency analysis for input intermediary, which corresponded to the Health Investment Efficiency (HIE). The input and intermediary were each composed of three variables. The second analysis was intermediary → output, which corresponded to the Health Competitiveness Efficiency (HCE), also each composed of three variables. The third analysis was conducted by the direct route of input → output to see the efficiency result of the overall investment, which was referred to as Overall Efficiency (OE).

Table 2 shows the average efficiency of healthcare and welfare investments in 34 Asian countries in our study.

Table 2 shows the mean values for the analysis results of 34 countries in three routes analysis. According to the results for each stage, the efficiency analysis result of Stage 1 corresponding to the Health Investment Efficiency (HIE) showed 0.399, and the efficiency analysis result of Stage 2 corresponding to the Health Competitiveness Efficiency (HCE) showed 0.612. Finally, the efficiency analysis result of Stage 3, which corresponds to the Overall Efficiency (OE), was 0.669, which showed the highest efficiency result among the three routes in terms of relative efficiency results. It should be noted that the estimated efficiency scores are in relative terms. Therefore, they should not be interpreted as absolute numbers in levels. Nevertheless, the closer they are to one, the higher is the relative efficiency compared to the other countries in the sample.

In Table 2, the value of the efficiency of Stage 2 is found to be relatively higher than Stage 1, which means that it takes more time and effort to establish the indicator related to healthcare providers in the intermediary from the input. On the other hand, Stage 2 shows that the intermediary variables of healthcare providers are more generally efficient in increasing the efficiency of output variables. Therefore, it is important to invest in the intermediary variable itself, but if the healthcare professionals and related industries, and medical technologies are developed together, the indicators of the “Health Care Indicators” corresponding to the output efficiency will be improved further.

Table 3 shows how the raw data values of each indicator in terms of time frames have changed as “time frame 1” → “time frame 2” → “time frame 3” progressively. Table 3 displays 34 countries’ average scores of input, intermediary, and output raw data by time frames. For example, the three indicators of output were gradually improving toward period_1 (2008–2010), period_2 (2011–2013), and period_3 (2014–2016). By indicator, first, “output 1” corresponding to an incidence of tuberculosis indicated that the incidence gradually decreased from period_1 (503.547), to period_2 (477.818), and to period_3 (453.024). Second, the “output 2” corresponding to the mortality rate, under-five showed that the mortality rate decreased from period_1 (110.600), to period_2 (96.715), and to period_3 (86.203). Third, “output 3”, which corresponds to life expectancy at birth, total (years), indicates that life expectancy increased from period_1 (70.098), to period_2 (70.851), and to period_3 (71.564). It is observed that some indicators in the input and intermediary do not increase proportionally to the time frame. Nevertheless, it is important to note that this may be attributed to various factors such as lack of sustained investments or lack of policy consistency in individual countries. Hence, it can be expected that the output efficiency may vary depending on the investment by item in accordance with national policy and may also change depending on the time frame.

Table 4 shows the top 10 countries with the highest values and bottom 10 countries that had the lowest values. Full results for all 34 countries are included in the Appendix A Table A1, Table A2, Table A3 and Table A4.

The efficiency scores were computed for three categories: Health Investment Efficiency (HIE), Health Competitiveness Efficiency (HCE), and Overall Efficiency (OE). Of the top ten scores for the highest “Health Investment Efficiency (HIE)” indicators, Kazakhstan3 (1.000000), India2 (1.000000), Kazakhstan2 (0.932438), and Azerbaijan2 (0.916659) show a relatively high efficiency. However, the efficiency scores of “Health Competitiveness Efficiency (HCE)” indicators for these four countries were overall low compared to the relatively high efficiency indicators of the “Health Investment Efficiency (HIE)”, and all four countries were in the bottom 10: Kazakhstan3 (0.141866), India2 (0.205447), Kazakhstan2 (0.167765), Azerbaijan2 (0.191514). This means that the efficiency indicators for the results of an intermediary for the given input are good, but the results of output for a given intermediary is relatively low.

As seen above, the differences with the other 34 countries were analyzed and compared based on the results of the high efficiency of Kazakhstan3, India2, Kazakhstan2, and Azerbaijan2 in the Health Investment Efficiency (HIE) sector.

Table 5 presents the results of a comparative analysis of the differences in these efficiency values.

Kazakhstan3 has higher investment indicators for “input 1” and “input 2” compared to the average of 34 countries. Intermediary indicators after three years show that the differences are more than ten times higher, indicating that the efficiency index of “Health Investment Efficiency (HIE)” is the highest. In particular, in Kazakhstan3, the value of the “Domestic General Government Health Expenditure (GGHE-D) as % Current Health Expenditure (CHE)” indicator, which corresponds to the “input 2” indicators, is 72.526, which is 74% higher than the average of 41.574 in the 34 countries. Thus, increasing weight in the government health expenditure sector is important for enhancing the “Health Investment Efficiency (HIE)” efficiency.

In terms of “Health Competitiveness Efficiency (HCE)”, “output 3” of Kazakhstan3 is similar to the average value of 34 countries, and “output 1” and “output 2” are 50% lower than the average of 34 countries, showing relatively superior performance. For reference, the indicators of “output 1” and “output 2” show the incidence of tuberculosis (per 100,000 people), mortality rate under-five (per 1000 live births), respectively. In contrast, we have seen that the efficiency in the “Health Competitiveness Efficiency (HCE)” of Kazakhstan3 (0.141866) is relatively low in Table 4 although the output indicators are superior to other countries in Table 5. The low “Health Competitiveness Efficiency (HCE)” may be because the number of “Health Care Providers” in the intermediary is more than adequate compared to the average of 34 countries. It could also be interpreted as a lack of competitive healthcare providers or inefficient healthcare settings and policies. It also suggests that it would be due to the differences in healthcare policies, and not just by healthcare providers, but also hospitals, systems, and the establishment of an efficient environment that are important items. As a reference, Kazakhstan has established policies that deviated from the former Soviet Union era in the 2000s. In particular, from 2005 to 2010, the country carried out national projects for the reorganization and development of the healthcare and welfare sector. Since 2010, many investments have been undertaken, focusing on improving the full-scale investment in the health and welfare policy and the expansion of medical benefits, while still striving to resolve health development and regional imbalances. There was an emphasis on developing new clinical guidelines [44].

Furthermore, since the 2000s, the Kazakhstan government has promoted the modernization of the healthcare sector by introducing the latest medical equipment or replacing clinic facilities with the help of a World Bank loan. More than 80% of Kazakhstan’s hospitals and health care institutions are state-owned, and in 2009, the country drafted health laws that meet international standards, with the intention of joining the WTO [45]. These investments in the healthcare and welfare sectors, as a result, show that the indicators of “intermediary 1”, “intermediary 2”, and “intermediary 3” corresponding to Kazakhstan3 are 10 times higher than the average of 34 countries. In addition to investing in the “Health Care Personnel”, there is also a focus on country-specific collaboration through MOUs such as health policies and systems, new medical technologies and devices, e-health, pharmaceutical industry, networks, and professional exchanges. Thus, if the skills and competitiveness in the “Health Care Personnel” field are increased through such continuous investments and collaborations, the efficiency indicator value for the “Health Competitiveness Efficiency (HCE)” may be improved. Above all, it is important to recognize the areas that need improvement as the example of Kazakhstan shows and implement policies from various perspectives to address the shortcomings and the inefficiencies.

In India2, indicators of “input 1” and “input 2” account for only 29% and 53%, respectively, which are relatively lower than the average for 34 countries. There is a potential for increasing investments in health expenditure, public health, and expanding the health care system. Additionally, the “output 1” and “output 2” indicators were 703.000 (147%) and 157.200 (163%), respectively, higher than the average of 34 countries. Unlike Kazakhstan3, it is important for India2 to establish a policy focusing on lowering the incidence of tuberculosis and under-five mortality rate.

India’s economy is already a global powerhouse and the industry is growing fast, but its health service sector performance is lower than average and vulnerable. For example, the top ten causes of death in India include premature birth complications, which are not highlighted in the OECD, but a major issue in India and in other low- and lower-middle income countries in Asia. As a result, India continues to draw attention and investment in insurance benefits which are being implemented through projects at a national level, which is one of the most important areas in terms of income and job creation in a country with a population of more than 1.3 billion.

In this context, the major initiatives taken by the government of India to push forward the Indian health care industry are as follows. The government of India launched Pradhan Mantri Jan Arogya Yojana (PMJAY) in 2018 to provide national health insurance of over 500,000 Rs (USD 7124) to more than 100 million families each year. In addition, India started Mission Indradhanush to improve the countries’ immunization coverage. It aims to achieve at least 90% vaccination coverage by 2018 [46].

The countries such as Kazakhstan3, India2, Kazakhstan3, and Azerbaijan2 included in the top 10 countries in Table 4 show higher levels of “Health Investment Efficiency (HIE)”, but in terms of “Health Competitiveness Efficiency (HCE)”, they show lower scores and are included in the bottom 10 countries. This can be interpreted as the environment, systems, and policies related to health care providers are inefficient or not managed properly. In this regard, it is necessary to increase the efficiency of the overall health and welfare policy by aiming to improve output indicators more directly. For example, Azerbaijan imports 98% of its medicines from other countries despite the high morbidity rate. Therefore, it will be necessary to invest in the healthcare and welfare systems of the economy [47]. In addition, relatively low levels of government expenditure on health as a proportion of the gross domestic product since independence has meant that out of pocket (OOP) payments accounted for almost 62% of total health expenditure in 2007. This has serious implications for access to care and financial risk [48].

Indonesia also shows relatively good efficiency indicators. Table 6 presents the detailed results on Indonesia.

It is significant that all three time frames for Indonesia are included among the Top 10 of Overall Efficiency (OE) results with Indonesia1 (0.977166), Indonesia2 (0.946518), and Indonesia3 (0.945692). The average value of variables corresponding to the inputs and outputs of Indonesia1, Indonesia2, and Indonesia3 indicate that most of the variables gradually increased or showed improved indicator values over time. These results can be inferred from the systematic and consistent investment in the healthcare sector in Indonesia and improved operationalization of the policy in the health and welfare sectors. However, investment in the “Domestic General Government Health Expenditure (GGHE-D)”, which corresponds to “input 2”, is still relatively lower than the average of 34 countries (32.471 vs. 39.773); hence, an increased focus on this area can further improve efficiency.

In contrast, countries with relatively low efficiency outcomes include Kuwait, Jordan, Maldives, Turkmenistan, and Afghanistan (Table 7). In particular, Kuwait showed relatively low efficiency values not only in “Overall Efficiency (OE)” but also in “Health Investment Efficiency (HIE)”, and “Health Competitiveness Efficiency (HCE)”.

The raw data values for Kuwait show that the “Current Health Expenditure (CHE)” value corresponding to “input 1” was about five to six times higher than the average of 34 countries, and the “Domestic General Government Health Expenditure (GGHE-D)” equivalent to “input 2” was also about two times higher. Therefore, Kuwait is showing generally low efficiency even though it spends a considerable amount of money on healthcare and makes relatively high investments in the public sector (Table 7). To improve these inefficiencies for Kuwait, the government should focus on improving the overall health care system and aim to increase the overall efficiency at the national level, keeping pace with consistent policies such as increasing the share of health insurance benefits. One suggestion is that Kuwait needs to have a competitive healthcare infrastructure through investments in human resources such as healthcare providers, technologies and investments in hospitals, and overall medical infrastructure.

Average levels of investments in health and welfare and the average levels of public health indicators in Asian economies are still low in a global comparison. The results on the measurement of relative efficiency in healthcare and welfare sectors of 34 Asian developing countries in our study identify key areas and indicators that need improvement in each country. Most importantly, it is essential to consistently invest in these areas and indicators with national and governmental goals. Benchmarking with more efficient countries’ indicators will also help in guiding the policies and programs in healthcare and welfare areas.

Asian developing countries are in a different situation than developed economies. Economic development indicators such as GDP are still growing at high rates in Asia and the health and medical industries are also growing in line with overall economic growth. Hence, the policy direction should be somewhat different in Asian developing countries compared to developed economies, e.g., OECD countries. It will be necessary to increase the efficiency of national healthcare and welfare policies by establishing relevant policies that are tailored to the current economic situation, characteristics, and external environments of the countries in question.

## 5. Conclusions

This study analyzes the characteristics and efficiency of the healthcare industry in 34 Asian countries by means of the two-stage DEA model derived from the existing traditional DEA model. Previous studies in the literature analyzed the efficiency through multi-output at multi-input models. Our two-stage DEA model includes input, intermediary, and output layers capturing the direction and the nature of the national healthcare and welfare policies in each country. The input, intermediary, and output layers are related to each other. This approach enables the results obtained to provide more specific and practical representation of the efficiency characteristics and changes in them overtime. Moreover, it is a meaningful approach to compare countries’ efficiency scores by time series, since it is a critical task for the country-level in the field of healthcare policy making. Countries need to consider how to supplement the policies and directions for the insufficient efficiency area. Therefore, complementation by comparison with other countries is essential, but it is also crucial to see how it fluctuated over time in the country itself.

As stated in the Introduction, the proposals and implementation of policies in healthcare and welfare areas constantly evolve globally as countries focus more attention on the healthcare industry to promote the health and welfare of their citizens. However, in the situation where an indicator setting and evaluation are needed to improve the results in terms of efficiency by focusing on each country’s efforts and investments, a formal format for evaluating the system has been lacking until now. This study not only analyzes the characteristics and efficiencies of national health and welfare policies in individual countries, but also applies the basic raw data as variables that can be commonly used to make comparisons between countries around the world in the field of healthcare and welfare. Before data selection, we investigated the real world’s essential indicators in the recent healthcare sector, which are currently being discussed globally and included related indicators for the analysis in this paper. The model employed in this study can be used as a basic framework for comparing the characteristics and efficiency analysis of the healthcare and welfare industry by country. The data sources are publicly available. At the national level, the data used in this study might be used to benchmark the strengths of other countries and to address the weakness of national policies and strengthen the policy-making process of the healthcare and welfare sectors. The methodological framework, the operationalization of different indicators of healthcare and welfare policies and their outcomes, and the findings of this study can be helpful in establishing future policies in the field of healthcare and welfare. The overall framework can play a major role in judging the efficiency of investments and identifying collaboration possibilities.

## 6. Limitations of the Study and Directions for Further Research

This study uses country-level data from 2002 to 2016 to analyze the characteristics and efficiency of national health and welfare policies. Some limitations exist in comparing the most recent policy data for 2019 because it usually takes lag to update common data for each country. Investment in the healthcare and welfare sectors continues to be made, and is a field of high interest in each country. Due to the nature of the efficiency analyses, the efficiency indicators in this study are only comparative values of relative efficiency. In other words, there is room for improvement even in countries with high efficiency indicators.

The variable set can also be expanded in future studies. Additional variables, such as “Primary Health Care (PHC) Expenditure per Capita” or “rate of national health insurance benefits” could be used in strategizing national policies as more and comparable cross-country data become available. In particular, the “Primary Health Care (PHC) Expenditure per Capita” category is a field that has attracted investments and interests all over the world and is one of the top priority areas for national health as part of chronic disease prevention projects. Many projects are necessary for the establishment and implementation of national policies in this area. Hence, an integrated approach to manage and prevent chronic diseases is essential in primary healthcare in low and middle-income countries [49]. Several clinical guidelines of medical societies also emphasize the importance of primary healthcare and prevention worldwide [50].

Currently, country-level data on “Primary Health Care (PHC) Expenditure per Capita” is not yet publicly available. When it becomes available, it would be an important additional insight to efficiency assessments of healthcare and welfare investments. The second potential variable to include in the model is the rate of national health insurance benefits. It may be used as an important indicator because it is also in line with the financial and economic conditions of the national health care sector. The addition of these two variables into the model will complement the results of previous studies that evaluate the relative achievements of national healthcare policy efficiency in individual countries.

Finally, although not directly linked to the healthcare and welfare sector, comparisons with sub-indicators such as the poverty headcount ratio at national poverty lines (% of the population) as indirect indicators will be meaningful in a more comprehensive and broader perspective. It may be used as a reference for the establishment and operation of healthcare and welfare policies. Overall, further studies on the subject would provide additional benchmarks in determining the direction of national policies.

## Figures and Tables

**Table 1:** Example of Afghanistan’s time frame (TF_1, TF_2, TF_3) for the analysis.

Time Frame	Country	Input Variable		Intermediary Variable		Output Variable	
TF_1	Afghanistan1	Input 1	2002–2004	Intermediary 1	2005–2007	Output 1	2008–2010
		Input 2		Intermediary 2		Output 2	
		Input 3		Intermediary 3		Output 3	
TF_2	Afghanistan2	Input 1	2005–2007	Intermediary 1	2008–2010	Output 1	2011–2013
		Input 2		Intermediary 2		Output 2	
		Input 3		Intermediary 3		Output 3	
TF_3	Afghanistan3	Input 1	2008–2010	Intermediary 1	2011–2013	Output 1	2014–2016
		Input 2		Intermediary 2		Output 2	
		Input 3		Intermediary 3		Output 3	

Input 1: Current Health Expenditure (CHE) per Capita in Purchasing Power Parity (PPP). Input 2: Domestic General Government Health Expenditure (GGHE-D) as % Current Health Expenditure (CHE). Input 3: Current Health Expenditure (CHE) as % Gross Domestic Product (GDP). Intermediary 1: Health personnel: Physicians (per 1000 population). Intermediary 2: Health personnel: Pharmacists (per 1000 population). Intermediary 3: Health personnel: Nurses and midwives personnel (per 1000 population). Output 1: Incidence of tuberculosis (per 100,000 people). Output 2: Mortality rate, under-five (per 1000 live births). Output 3: Life expectancy at birth, total (years).

**Table 2:** Average efficiency of 34 countries in Asia.

Country	^1^ HIE_Stage 1	^2^ HCE_Stage 2	^3^ OE_Stage 3
Average of 34 countries	0.399	0.612	0.669

^1^ HIE (Health Investment Efficiency): Input → Intermediary. ^2^ HCE (Health Competitiveness Efficiency): Intermediary → Output. ^3^ OE (Overall Efficiency): Input → Output.

**Table 3:** Average values of input, intermediary, and output raw data by time frame in 34 countries.

Input	Average of Raw Data	Intermediary	Average of Raw Data	Output	Average of Raw Data
Input 1 (2002–2004)	1064.593	Intermediary 1 (2005–2007)	0.412	Output 1 (2008–2010)	503.547
Input 1 (2005–2007)	1203.549	Intermediary 1 (2008–2010)	1.365	Output 1 (2011–2013)	477.818
Input 1 (2008–2010)	1455.074	Intermediary 1 (2011–2013)	0.229	Output 1 (2014–2016)	453.024
Input 2 (2002–2004)	38.819	Intermediary 2 (2005–2007)	0.141	Output 2 (2008–2010)	110.600
Input 2 (2005–2007)	38.926	Intermediary 2 (2008–2010)	0.300	Output 2 (2008–2010)	96.715
Input 2 (2008–2010)	41.574	Intermediary 2 (2011–2013)	0.074	Output 2 (2008–2010)	86.203
Input 3 (2002–2004)	4.775	Intermediary 3 (2005–2007)	0.715	Output 3 (2008–2010)	70.098
Input 3 (2005–2007)	4.563	Intermediary 3 (2008–2010)	2.685	Output 3 (2008–2010)	70.851
Input 3 (2008–2010)	4.614	Intermediary 3 (2011–2013)	0.509	Output 3 (2008–2010)	71.564

Input 1: Current Health Expenditure (CHE) per Capita in PPP. Input 2: Domestic General Government Health Expenditure (GGHE-D) as % Current Health Expenditure (CHE). Input 3: Current Health Expenditure (CHE) as % Gross Domestic Product (GDP). Intermediary 1: Health personnel: Physicians (per 1000 population). Intermediary 2: Health personnel: Pharmacists (per 1000 population). Intermediary 3: Health personnel: Nurses and midwives personnel (per 1000 population). Output 1: Incidence of tuberculosis (per 100,000 people). Output 2: Mortality rate, under-five (per 1000 live births). Output 3: Life expectancy at birth, total (years).

**Table 4:** Healthcare and welfare efficiency scores by top 10 and bottom 10 countries.

Country		Hie (Health Investment Efficiency)		Hce (Health Competitiveness Efficiency)		Oe (Overall Efficiency)	
		Country	Efficiency Score	Country	Efficiency Score	Country	Efficiency Score
Top 10 countries	1	Kazakhstan3	1.000000	Philippines1	1.000000	Myanmar1	1.000000
	2	India2	1.000000	Armenia1	1.000000	Bangladesh2	1.000000
	3	Kazakhstan2	0.932438	Georgia1	1.000000	Indonesia1	0.977166
	4	Azerbaijan2	0.916659	Malaysia1	1.000000	Indonesia2	0.946518
	5	Tajikistan2	0.861501	Kazakhstan1	1.000000	Indonesia3	0.945692
	6	Myanmar3	0.837092	Thailand1	1.000000	Nepal2	0.903005
	7	Syria2	0.835289	Iran2	1.000000	China1	0.892908
	8	Mongolia3	0.811569	Mongolia1	1.000000	India2	0.889808
	9	Philippines3	0.805536	Maldives1	1.000000	Tajikistan1	0.884098
	10	Mongolia2	0.794839	Kuwait1	1.000000	Philippines1	0.874944
Bottom 10 countries	10	Thailand1	0.152892	India2	0.205447	Jordan1	0.471574
	9	Iran2	0.151942	Oman2	0.203672	Jordan2	0.464351
	8	Lebanon3	0.150029	Jordan1	0.195256	Afghanistan2	0.459803
	7	Mongolia1	0.148295	Lebanon2	0.193653	Turkmenistan1	0.447963
	6	Maldives1	0.134348	Azerbaijan2	0.191514	Kuwait2	0.438198
	5	Turkmenistan1	0.132866	Turkmenistan2	0.186355	Jordan3	0.419893
	4	Jordan3	0.123249	Jordan2	0.176407	Kuwait1	0.409494
	3	Kuwait3	0.121946	Kazakhstan2	0.167765	Afghanistan1	0.393866
	2	Maldives3	0.111879	Kuwait2	0.160848	Kuwait3	0.393410
	1	Kuwait1	0.094220	Kazakhstan3	0.141866	Maldives3	0.382635

The above efficiency scores indicate relative efficiency. Hence, even if a country shows 1.000000 score, it does not mean that the country has the globally best efficiency. It is relatively efficient than other countries but there are still areas that can be improved.

**Table 5:** Comparison of the average values of input, intermediary, and output variables between the four selected countries and 34 countries.

Country	Input 1	Input 2	Input 3	Intermediary 1	Intermediary 2	Intermediary 3	Output 1	Output 2	Output 3
^1^ Average of 34 countries (TF_3)	1455.074	41.574	4.614	0.229	0.074	0.509	453.024	86.203	71.564
Kazakhstan3	1723.213	72.526	3.085	3.500	0.800	8.500	243.000	36.100	71.973
Ratio a	118%	174%	67%	1526%	1088%	1671%	54%	42%	101%
^2^ Average of 34 countries (TF_2)	1203.549	38.926	4.563	1.365	0.300	2.685	477.818	96.715	70.851
India2	354.734	20.511	3.648	1.300	1.000	2.900	703.000	157.200	67.368
Ratio b	29%	53%	80%	95%	333%	108%	147%	163%	95%
Kazakhstan2	1543.589	66.096	3.329	3.500	0.700	8.300	359.000	49.400	69.680
Ratio c	128%	170%	73%	256%	233%	309%	75%	51%	98%
Azerbaijan2	1655.464	15.706	5.954	3.600	0.200	8.000	289.000	98.000	71.465
Ratio d	138%	40%	130%	264%	67%	298%	60%	101%	101%

^1, 2^ The mean value was calculated as the average value for the period connected to the country time frame. Ratio a: Kazakhstan3/Average value; Ratio b: India2/Average value; Ratio c: Kazakhstan2/Average value; Ratio d: Azerbaijan2/Average value. Input 1: Current Health Expenditure (CHE) per Capita in PPP. Input 2: Domestic General Government Health Expenditure (GGHE-D) as % Current Health Expenditure (CHE). Input 3: Current Health Expenditure (CHE) as % Gross Domestic Product (GDP). Intermediary 1: Health personnel: Physicians (per 1000 population). Intermediary 2: Health personnel: Pharmacists (per 1000 population). Intermediary 3: Health personnel: Nurses and midwives personnel (per 1000 population). Output 1: Incidence of tuberculosis (per 100,000 people). Output 2: Mortality rate, under-five (per 1000 live births). Output 3: Life expectancy at birth, total (years).

**Table 6:** Average value of the variable in Indonesia.

Country	Input 1	Input 2	Input 3	Intermediary 1	Intermediary 2	Intermediary 3	Output 1	Output 2	Output 3
Indonesia1	355.736	34.328	2.176	0.000	0.000	0.000	1036.000	104.300	67.963
Indonesia2	544.553	31.615	2.711	1.100	0.000	0.100	1005.000	91.800	68.508
Indonesia3	656.242	31.469	2.753	1.400	0.000	0.200	976.000	81.800	69.024

**Table 7:** Comparison of average values between Kuwait and 34 countries.

Country	Input 1	Input 2	Input 3	Intermediary 1	Intermediary 2	Intermediary 3	Output 1	Output 2	Output 3
^3^ Average of 34 countries (TF_1)	1064.593	38.819	4.775	0.412	0.141	0.715	503.547	110.600	70.098
Kuwait1	6305.675	79.894	3.188	0.000	0.000	0.000	113.000	33.200	73.870
Ratio e	592%	206%	67%	0%	0%	0%	22%	30%	105%
^4^ Average of 34 countries (TF_2)	1203.549	38.926	4.563	1.365	0.300	2.685	477.818	96.715	70.851
Kuwait2	5915.978	79.862	2.255	4.300	0.300	11.100	71.000	29.900	74.217
Ratio f	492%	205%	49%	315%	100%	413%	15%	31%	105%
^5^ Average of 34 countries (TF_3)	1455.074	41.574	4.614	0.229	0.074	0.509	453.024	86.203	71.564
Kuwait3	6841.809	83.322	2.853	0.000	0.000	0.000	68.000	26.300	74.576
Ratio g	470%	200%	62%	0%	0%	0%	15%	31%	104%

^3, 4, 5^ The mean value was calculated as the average value for the period connected to the country time frame. Ratio e: Kuwait1/Average value; Ratio f: Kuwait2/Average value; Ratio g: Kuwait3/Average value.

## Appendix A

**Table A1:** List of countries and the data used in the study (Time Frame_1).

Country (TF_1)	Input 1	Input 2	Input 3	Int. 1	Int. 2	Int. 3	Output 1	Output 2	Output 3
Afghanistan1	248.347	4.569	9.398	0.000	0.000	0.600	567.000	281.800	60.741
Armenia1	701.800	20.630	6.510	0.000	0.000	0.000	200.000	57.000	73.132
Azerbaijan1	854.045	16.248	5.685	0.000	0.000	0.000	345.000	120.100	70.624
Bahrain1	4108.647	64.707	3.772	1.100	0.200	2.800	86.000	26.700	75.913
Bangladesh1	104.253	24.717	2.186	0.300	0.000	0.300	663.000	157.100	69.756
Bhutan1	430.305	69.209	4.309	0.200	0.100	0.800	593.000	135.700	67.299
Cambodia1	294.087	20.180	7.099	0.000	0.000	0.000	1351.000	144.400	65.985
China1	513.337	28.001	4.328	0.000	0.300	1.000	242.000	51.300	75.025
Georgia1	792.838	13.442	8.206	0.000	0.000	0.000	400.000	53.600	72.657
India1	295.055	18.288	4.069	0.600	0.000	1.300	762.000	184.700	66.215
Indonesia1	355.736	34.328	2.176	0.000	0.000	0.000	1036.000	104.300	67.963
Iran1	1873.741	38.368	5.025	0.900	0.200	1.400	50.000	62.000	73.486
Iraq1	536.726	58.131	3.417	0.000	0.000	0.000	137.000	113.300	68.308
Jordan1	1879.090	44.436	9.265	2.400	1.300	3.300	19.600	65.100	73.250
Kazakhstan1	1270.397	55.091	3.771	0.000	0.000	0.000	452.000	67.800	67.916
Kuwait1	6305.675	79.894	3.188	0.000	0.000	0.000	113.000	33.200	73.870
Lao P D Republic1	310.417	24.820	3.906	0.300	0.000	1.000	691.000	250.000	63.884
Lebanon1	2645.785	34.626	8.573	0.000	1.000	1.200	43.000	32.500	78.161
Malaysia1	1346.211	51.505	2.875	0.000	0.000	0.000	221.000	23.300	74.028
Maldives1	1947.931	30.533	7.682	0.000	0.000	0.000	111.000	43.500	75.893
Mongolia1	624.367	64.012	4.049	0.000	0.000	0.000	1284.000	86.700	66.917
Myanmar1	109.810	11.581	2.087	0.400	0.000	0.700	1164.000	220.400	64.839
Nepal1	182.395	17.746	4.502	0.000	0.000	0.000	491.000	149.100	67.478
Oman1	3341.948	82.752	3.110	1.700	0.700	3.700	40.000	35.200	75.444
Pakistan1	251.116	30.251	2.537	0.800	0.000	0.000	828.000	278.400	64.848
Philippines1	343.168	36.327	3.031	0.000	0.000	0.000	1583.000	95.600	68.212
Sri Lanka1	693.253	53.745	4.039	1.100	0.000	3.500	198.000	35.800	74.276
Syria1	533.411	47.005	4.707	1.600	0.700	1.900	69.000	48.800	72.898
Tajikistan1	177.446	17.742	4.646	2.000	0.000	0.000	424.000	134.900	69.279
Thailand1	860.825	65.746	3.239	0.000	0.000	0.000	565.000	42.000	73.606
Timor-Leste1	106.567	44.569	2.196	0.000	0.000	0.000	1494.000	196.000	66.914
Turkmenistan1	1244.795	29.798	8.700	0.000	0.000	0.000	253.000	184.400	66.348
Viet Nam1	368.783	45.034	4.683	0.600	0.300	0.800	478.000	69.600	74.947
Yemen1	543.864	41.803	5.368	0.000	0.000	0.000	167.000	176.100	63.201

Input 1: Current Health Expenditure (CHE) per Capita in PPP. Input 2: Domestic General Government Health Expenditure (GGHE-D) as % Current Health Expenditure (CHE). Input 3: Current Health Expenditure (CHE) as % Gross Domestic Product (GDP). Intermediary 1: Health personnel: Physicians (per 1000 population). Intermediary 2: Health personnel: Pharmacists (per 1000 population). Intermediary 3: Health personnel: Nurses and midwives personnel (per 1000 population). Output 1: Incidence of tuberculosis (per 100,000 people). Output 2: Mortality rate, under-five (per 1000 live births). Output 3: Life expectancy at birth, total (years).

**Table A2:** List of countries and the data used in the study (Time Frame_2).

Country (TF_2)	Input 1	Input 2	Input 3	Int. 1	Int. 2	Int. 3	Output 1	Output 2	Output 3
Afghanistan2	314.541	5.220	10.159	0.600	0.000	1.100	567.000	248.000	62.082
Armenia2	1008.572	26.375	6.023	2.800	0.000	5.500	168.000	48.900	73.810
Azerbaijan2	1655.464	15.706	5.954	3.600	0.200	8.000	289.000	98.000	71.465
Bahrain2	3751.796	67.360	3.181	0.900	0.200	2.400	58.000	24.000	76.341
Bangladesh2	139.884	22.718	2.328	0.400	0.400	0.200	663.000	130.900	71.032
Bhutan2	516.182	61.450	4.114	0.200	0.100	1.000	572.000	114.600	68.635
Cambodia2	348.524	17.756	5.958	0.200	0.000	0.900	1234.000	114.900	67.456
China2	688.331	35.641	3.907	1.500	0.300	1.500	220.000	40.600	75.599
Georgia2	1116.971	15.021	7.931	4.300	0.100	4.000	357.000	43.600	72.751
India2	354.734	20.511	3.648	1.300	1.000	2.900	703.000	157.200	67.368
Indonesia2	544.553	31.615	2.711	0.100	0.000	1.100	1005.000	91.800	68.508
Iran2	2363.817	39.458	5.181	0.000	0.000	0.000	54.000	54.100	74.772
Iraq2	880.057	67.247	2.809	0.600	0.200	0.000	135.000	105.200	68.961
Jordan2	2113.986	53.230	8.407	2.500	1.400	3.900	15.800	59.600	73.728
Kazakhstan2	1543.589	66.096	3.329	3.500	0.700	8.300	359.000	49.400	69.680
Kuwait2	5915.978	79.862	2.255	4.300	0.300	11.100	71.000	29.900	74.217
Lao P D Republic2	322.244	22.388	3.141	0.200	0.000	0.900	614.000	225.200	65.201
Lebanon2	2899.019	40.178	8.248	2.700	1.300	2.000	42.000	28.200	78.855
Malaysia2	1693.005	51.934	3.046	1.200	0.400	3.200	257.000	22.500	74.604
Maldives2	2505.561	39.175	8.394	1.600	0.700	5.600	101.000	32.700	76.410
Mongolia2	640.384	63.234	2.958	2.800	0.400	3.600	1284.000	67.600	68.198
Myanmar2	152.565	9.717	1.893	0.500	0.000	0.900	1129.000	177.000	65.792
Nepal2	200.941	20.478	4.246	0.000	0.000	0.000	482.000	127.500	68.729
Oman2	2839.504	81.391	2.441	2.000	0.900	4.400	35.000	34.200	76.140
Pakistan2	346.313	19.806	2.951	0.900	0.000	0.600	827.000	259.700	65.677
Philippines2	536.247	32.169	3.922	0.000	0.000	0.000	1617.000	91.300	68.555
Sri Lanka2	866.631	50.700	3.980	0.700	0.000	1.800	198.000	32.400	74.599
Syria2	510.117	48.216	3.870	1.500	0.800	1.900	57.000	51.500	70.622
Tajikistan2	259.446	19.010	5.261	1.700	0.000	4.500	325.000	118.700	70.223
Thailand2	1045.564	73.400	3.151	0.400	0.100	2.100	518.000	36.300	74.440
Timor-Leste2	189.592	32.457	1.143	0.100	0.100	1.200	1494.000	171.900	67.805
Turkmenistan2	1492.071	21.913	7.752	2.300	0.200	4.800	207.000	167.300	67.157
Viet Nam2	530.361	42.690	5.317	0.700	0.300	1.200	442.000	67.300	75.478
Yemen2	634.132	29.354	5.519	0.300	0.100	0.700	146.000	166.300	64.041

Input 1: Current Health Expenditure (CHE) per Capita in PPP. Input 2: Domestic General Government Health Expenditure (GGHE-D) as % Current Health Expenditure (CHE). Input 3: Current Health Expenditure (CHE) as % Gross Domestic Product (GDP). Intermediary 1: Health personnel: Physicians (per 1000 population). Intermediary 2: Health personnel: Pharmacists (per 1000 population). Intermediary 3: Health personnel: Nurses and midwives personnel (per 1000 population). Output 1: Incidence of tuberculosis (per 100,000 people). Output 2: Mortality rate, under-five (per 1000 live births). Output 3: Life expectancy at birth, total (years).

**Table A3:** List of countries and the data used in the study (Time Frame_3).

Country (TF_3)	Input 1	Input 2	Input 3	Int. 1	Int. 2	Int. 3	Output 1	Output 2	Output 3
Afghanistan3	403.725	5.671	9.548	0.000	0.000	0.000	567.000	219.500	63.285
Armenia3	957.844	35.029	4.767	0.000	0.000	0.000	151.000	42.000	74.439
Azerbaijan3	2122.185	22.803	4.765	0.000	0.000	0.000	213.000	79.400	71.916
Bahrain3	4458.306	64.313	3.728	0.000	0.000	0.000	43.000	22.800	76.761
Bangladesh3	176.008	20.907	2.415	0.000	0.000	0.000	663.000	109.300	72.149
Bhutan3	555.947	71.676	3.412	0.000	0.000	0.000	503.000	100.200	69.815
Cambodia3	503.836	17.096	7.032	0.000	0.000	0.000	1103.000	96.200	68.617
China3	1033.435	48.609	4.136	0.000	0.000	0.000	198.000	32.400	76.092
Georgia3	1632.930	20.385	9.354	0.000	0.000	0.000	297.000	36.300	73.103
India3	418.386	24.815	3.424	0.000	0.000	0.000	651.000	132.400	68.294
Indonesia3	656.242	31.469	2.753	0.200	0.000	1.400	976.000	81.800	69.024
Iran3	3130.786	35.362	6.199	0.000	0.000	0.000	46.000	48.100	75.716
Iraq3	1207.450	76.634	3.345	0.000	0.000	0.000	129.000	96.800	69.664
Jordan3	2492.224	64.768	8.882	0.000	0.000	0.000	16.800	54.400	74.182
Kazakhstan3	1723.213	72.526	3.085	3.500	0.800	8.500	243.000	36.100	71.973
Kuwait3	6841.809	83.322	2.853	0.000	0.000	0.000	68.000	26.300	74.576
Lao P D Republic3	380.943	24.131	3.047	0.000	0.000	0.000	546.000	203.600	66.331
Lebanon3	3360.549	39.649	7.408	0.000	0.000	0.000	39.000	25.200	79.408
Malaysia3	1976.736	53.838	3.211	0.000	0.000	0.000	272.000	22.800	75.140
Maldives3	3527.119	55.478	10.017	0.000	0.000	0.000	134.000	26.400	77.055
Mongolia3	821.199	64.319	3.036	2.900	0.500	3.700	1284.000	56.600	69.074
Myanmar3	200.097	8.770	1.947	0.600	0.000	0.900	1095.000	157.300	66.454
Nepal3	256.674	19.058	4.593	0.500	0.200	1.600	468.000	109.900	69.884
Oman3	3337.708	80.948	2.529	0.000	0.000	0.000	28.000	33.700	76.803
Pakistan3	337.860	20.994	2.711	0.000	0.000	0.000	808.000	238.500	66.314
Philippines3	664.645	30.897	4.231	0.000	0.900	0.000	1650.000	87.300	68.953
Sri Lanka3	1030.624	41.419	4.015	0.000	0.000	0.000	195.000	28.600	75.093
Syria3	505.735	45.270	3.407	0.000	0.000	0.000	63.000	52.200	70.010
Tajikistan3	345.461	20.756	5.834	0.000	0.000	0.000	262.000	107.400	70.873
Thailand3	1313.525	76.641	3.490	0.000	0.000	0.000	490.000	31.400	75.100
Timor-Leste3	301.595	48.003	1.371	0.100	0.100	1.200	1494.000	153.500	68.578
Turkmenistan3	1419.288	21.443	5.238	0.000	0.000	0.000	153.000	151.500	67.697
Viet Nam3	670.442	42.438	5.469	0.000	0.000	0.000	410.000	64.800	76.053
Yemen3	707.985	24.068	5.636	0.000	0.000	0.000	144.000	166.200	64.740

Input 1: Current Health Expenditure (CHE) per Capita in PPP. Input 2: Domestic General Government Health Expenditure (GGHE-D) as % Current Health Expenditure (CHE). Input 3: Current Health Expenditure (CHE) as % Gross Domestic Product (GDP). Intermediary 1: Health personnel: Physicians (per 1000 population). Intermediary 2: Health personnel: Pharmacists (per 1000 population). Intermediary 3: Health personnel: Nurses and midwives personnel (per 1000 population). Output 1: Incidence of tuberculosis (per 100,000 people). Output 2: Mortality rate, under-five (per 1000 live births). Output 3: Life expectancy at birth, total (years).

**Table A4:** The efficiency estimates for all 34 countries (Time Frame_1, 2, 3).

Country	HIE_ Stage 1 (Health Investment Efficiency)	HCE_ Stage 2 (Health Competitiveness Efficiency)	OE_ Stage 3 (Overall Efficiency)
	Efficiency Score (Input → Intermediary)	Efficiency Score (Intermediary → Output)	Efficiency Score (Input → Output)
Afghanistan1	0.179372	0.580152	0.393866
Afghanistan2	0.250496	0.463905	0.459803
Afghanistan3	0.243516	0.561008	0.486654
Armenia1	0.188748	1.000000	0.762701
Armenia2	0.643430	0.286060	0.731398
Armenia3	0.254976	0.837924	0.765761
Azerbaijan1	0.207980	0.956082	0.754376
Azerbaijan2	0.916659	0.191514	0.695810
Azerbaijan3	0.249371	0.774038	0.695427
Bahrain1	0.309209	0.429717	0.504646
Bahrain2	0.287332	0.466794	0.524639
Bahrain3	0.157309	0.882301	0.502865
Bangladesh1	0.387652	0.715186	0.526539
Bangladesh2	0.603828	0.467351	1.000000
Bangladesh3	0.425754	0.737158	0.581456
Bhutan1	0.219310	0.628664	0.527021
Bhutan2	0.235989	0.628870	0.585021
Bhutan3	0.206950	0.762345	0.578940
Cambodia1	0.186318	0.911336	0.650614
Cambodia2	0.270786	0.723485	0.775593
Cambodia3	0.251876	0.764776	0.710459
China1	0.378402	0.615274	0.892908
China2	0.538262	0.396234	0.848563
China3	0.221638	0.875569	0.721108
Georgia1	0.170703	1.000000	0.708993
Georgia2	0.748990	0.234633	0.700777
Georgia3	0.179522	0.880323	0.590384
India1	0.420882	0.514167	0.832572
India2	1.000000	0.205447	0.889808
India3	0.319954	0.721877	0.864917
Indonesia1	0.255831	0.989876	0.977166
Indonesia2	0.329189	0.740503	0.946518
Indonesia3	0.435616	0.572582	0.945692
Iran1	0.342342	0.487786	0.646097
Iran2	0.151942	1.000000	0.614447
Iran3	0.170678	0.869835	0.570465
Iraq1	0.169639	0.974881	0.641723
Iraq2	0.279257	0.558542	0.608930
Iraq3	0.174241	0.804080	0.540087
Jordan1	0.622217	0.195256	0.471574
Jordan2	0.670814	0.176407	0.464351
Jordan3	0.123249	0.878978	0.419893
Kazakhstan1	0.157679	1.000000	0.642986
Kazakhstan2	0.932438	0.167765	0.608507
Kazakhstan3	1.000000	0.141866	0.579401
Kuwait1	0.094220	1.000000	0.409494
Kuwait2	0.713662	0.160848	0.438198
Kuwait3	0.121946	0.898844	0.393410
Lao P D Republic1	0.329707	0.522112	0.662726
Lao P D Republic2	0.390282	0.536688	0.790300
Lao P D Republic3	0.338448	0.623734	0.753888
Lebanon1	0.360734	0.369529	0.514827
Lebanon2	0.678649	0.193653	0.498423
Lebanon3	0.150029	0.911588	0.495041
Malaysia1	0.170312	1.000000	0.752818
Malaysia2	0.574866	0.328640	0.709799
Malaysia3	0.191126	0.938857	0.647739
Maldives1	0.134348	1.000000	0.578690
Maldives2	0.597532	0.225532	0.505589
Maldives3	0.111879	0.946208	0.382635
Mongolia1	0.148295	1.000000	0.596113
Mongolia2	0.794839	0.224304	0.665032
Mongolia3	0.811569	0.207325	0.601636
Myanmar1	0.535928	0.572696	1.000000
Myanmar2	0.752686	0.498871	0.693672
Myanmar3	0.837092	0.453869	0.616385
Nepal1	0.242074	0.928685	0.871043
Nepal2	0.298224	0.819634	0.903005
Nepal3	0.685321	0.376892	0.854013
Oman1	0.447170	0.281217	0.488601
Oman2	0.706680	0.203672	0.534916
Oman3	0.183312	0.859128	0.507564
Pakistan1	0.373875	0.466587	0.678171
Pakistan2	0.583700	0.363226	0.778804
Pakistan3	0.428729	0.555327	0.764069
Philippines1	0.219631	1.000000	0.874944
Philippines2	0.263650	0.857364	0.829435
Philippines3	0.805536	0.299240	0.781190
Sri Lanka1	0.388474	0.468811	0.711751
Sri Lanka2	0.377496	0.525870	0.731983
Sri Lanka3	0.319852	0.807393	0.768124
Syria1	0.592745	0.307295	0.714269
Syria2	0.835289	0.246034	0.762053
Syria3	0.339649	0.801677	0.813694
Tajikistan1	0.518253	0.435506	0.884098
Tajikistan2	0.861501	0.266721	0.838929
Tajikistan3	0.355619	0.730536	0.783234
Thailand1	0.152892	1.000000	0.666162
Thailand2	0.364042	0.484176	0.633431
Thailand3	0.234823	0.855042	0.613812
Timor-Leste1	0.225223	0.832965	0.735827
Timor-Leste2	0.621845	0.466882	0.544364
Timor-Leste3	0.568697	0.468536	0.807044
Turkmenistan1	0.132866	0.857006	0.447963
Turkmenistan2	0.781743	0.186355	0.527314
Turkmenistan3	0.372328	0.644576	0.682869
Viet Nam1	0.349586	0.519747	0.715061
Viet Nam2	0.523507	0.377083	0.700412
Viet Nam3	0.331494	0.754766	0.712775
Yemen1	0.161700	0.872370	0.557511
Yemen2	0.399160	0.452040	0.640019
Yemen3	0.359585	0.690045	0.701695

HIE (Health Investment Efficiency): Input → Intermediary. HCE (Health Competitiveness Efficiency): Intermediary → Output. OE (Overall Efficiency): Input → Output.
